# Activity of Fosfomycin- and Daptomycin-Containing Bone Cement on Selected Bacterial Species Being Associated with Orthopedic Infections

**DOI:** 10.1155/2017/2318174

**Published:** 2017-04-06

**Authors:** Sigrun Eick, Kevin Hofpeter, Anton Sculean, Claudia Ender, Susann Klimas, Sebastian Vogt, Sandor Nietzsche

**Affiliations:** ^1^Department of Periodontology, Laboratory of Oral Microbiology, School of Dental Medicine, University of Bern, Freiburgstrasse 7, 3010 Bern, Switzerland; ^2^Center of Electron Microscopy, University Hospital of Jena, Ziegelmühlenweg 1, 07743 Jena, Germany; ^3^Heraeus Medical GmbH, Philipp-Reis-Str. 8/13, 61273 Wehrheim, Germany

## Abstract

The purpose of this study was to determine activity of fosfomycin/gentamicin and daptomycin/gentamicin-containing PMMA bone-cement against* Staphylococcus aureus* (MRSA, MSSA),* Staphylococcus epidermidis*,* Enterococcus faecium *(VRE), and* E. coli* (ESBL; only fosfomycin). Test specimens of the bone cement were formed and bacteria in two concentrations were added one time or repeatedly up to 96 h. All fosfomycin-containing cement killed ultimately all MSSA,* Staphylococcus epidermidis,* and* E. coli* within 24 h; growth of MRSA was suppressed up to 48 h. Activity of daptomycin-containing cement depended on the concentration; the highest concentrated bone cement used (1.5 g daptomycin/40 g of powder) was active against all one-time added bacteria. When bacteria were added repeatedly to fosfomycin-containing cement, growth was suppressed up to 96 h and that of MRSA and VRE only up to 24 h. The highest concentration of daptomycin suppressed the growth of repeated added bacteria up to 48 h (VRE) until 96 h (MSSA, MRSA). In conclusion, PMMA bone cement with 1.5 g of daptomycin and 0.5 g of gentamicin may be an alternative in treatment of periprosthetic infections caused by Gram-positive bacteria.

## 1. Introduction

Prosthetic joint infections are a serious complication in arthroplastic surgery. A retrospective analysis of 450 clinics in the Western part of Germany by using a questionnaire revealed a prevalence of about 5-6% of septic arthroplastic revisions related to the numbers of implant placements [1]. In USA, the Nationwide Inpatient Sample (Q4 2005–2010) was analyzed for more than 200 thousand revisions of total hip arthroplasties (THA) and for more than 300 thousand revisions of total knee arthroplasties (TKA); periprosthetic joint infection was the most common reason for revision of TKA (25%) and the third most common reason for revision of THA (15.4%) [2].

Prosthetic joint infections are mainly caused by Gram-positive bacteria. In a recent study 33 clinical isolates were analyzed; most staphylococci being resistant against methicillin were identified [3]. Another study including 302 joints of 272 patients found in each about 40–45% infections caused by methicillin-sensitive and methicillin-resistant Gram-positive species only and about 15% where Gram-negative species (most prevalent* Escherichia coli*) were identified [4]. Infections are biofilm-associated. Bacteria undergo a transition from free-living, planktonic cells to sessile, surface-attached cells; the biologic cycle includes initiation, maturation, maintenance, and dissolution [5]. Aggregates of bacteria are embedded in a self-produced polymer matrix and are tolerant against innate and adaptive immune response as well as against antibiotics being active against planktonic bacteria [6].

The treatment of prosthetic joint infections is mostly a two-stage revision by using polymethylmethacrylate (PMMA) bone cement as spacer [1, 2]. This standard procedure for managing of infected TKAs and THAs consists of debridement with hardware removal, local and systemic antibiotic therapy, and delayed reimplantation [7]. The added antimicrobials should exhibit good antibacterial activity as well as not interfering negatively with osteointegration [7]. Addition of gentamicin to PMMA has been proven since the 70s of the last century [8, 9]. Later erythromycin alone or combined with colistin [10] and tobramycin [11] was applied. The raising development of resistance implicated the search for new agents. Silver nanoparticles were proved [12]. Among antibiotics, vancomycin is used and exerts a sufficient activity in joint fluid against methicillin-resistant* S. aureus* (MRSA) and coagulase-negative staphylococci [13].

The aim of this study was to determine activity of fosfomycin/gentamicin and daptomycin/gentamicin-containing bone cement against selected bacterial species to be known as potential infectious agents in peri-implant orthopedic infections. Fosfomycin and daptomycin were combined with gentamicin. The chosen bacterial strains were defined strains and exhibited mainly a high degree of resistance. Experiments were carried out up to 96 h with a daily removal of the spent media and an addition of limited amount of a fresh one simulating a decreasing amount of wound fluid. Beside the determination of the antimicrobial activity of the eluates the antibiofilm activity of the bone cement specimens was proven.

## 2. Materials and Methods

### 2.1. Microorganisms

The activity of the bone cement and their eluates was tested against two* Staphylococcus aureus* strains (*S. aureus* DSM 2569 = ATCC 29213 (MSSA),* S. aureus* DSM 13661 = ATCC 43300 (MRSA)),* Staphylococcus epidermidis* DSM 8913,* Enterococcus faecium *DSM 13590 (VRE), and* E. coli* DSM 22311 (ESBL:SHV-1). All the strains were obtained from the Leibniz Institute DSMZ German Collection of Microorganisms and Cell Culture, Braunschweig, Germany, being one of the largest biological resource centers worldwide.* S. aureus* ATCC 29212 and* S. aureus* ATCC 43300 are reference strains for testing antimicrobial resistance;* S. epidermidis* DSM 8913 was deposited as an isolate of a prosthetic hip infection, and* E. faecium *DSM 13590 and* E. coli* DSM 22311 were chosen because of their defined resistance mechanisms to selected antibiotics. Because of the natural resistance of* E. coli* to daptomycin, the daptomycin/gentamicin-containing cement was excluded from tests with that strain.

The strains were precultivated on tryptic-soy- (TS-) agar (Oxoid, Basingstoke, GB). The overnight culture was adjusted to 10^7^ bacteria/ml.

### 2.2. MIC Determination and Determination of Inhibition Zones

The MIC values of gentamicin, fosfomycin, and daptomycin against the included bacterial strains were determined by the microbroth dilution technique according to the EUCAST recommendations [14] in independent replicates.

To relate the antimicrobial activity of the bone cement eluates to those of the pure antibiotics, inhibition zones of the single antibiotics and in certain mixtures against the used bacterial strains were measured in addition. Dilution series of the antibiotics (Sigma-Aldrich Chemie GmbH, Buchs, Switzerland) were prepared from 3.13 mg/ml fosfomycin and from 1.56 mg/l daptomycin and gentamicin. The mixtures started from 1.56 mg/ml, 2.34 mg/ml, and 3.12 mg/ml fosfomycin and from 0.78 mg/ml, 1.56 mg/ml, and 2.34 mg/ml daptomycin each mixed with 0.78 mg/ml gentamicin. The calculation was based on the final concentration of antibiotics in bone cement related to 10 ml of elution media. Each 100 *µ*l of bacterial suspension (McFarland 0.5) was spread on Mueller-Hinton agar plates (Oxoid). Meanwhile, 10 *µ*l of the antibiotic solution was pipetted on a test disk (Oxoid) which was placed on the agar plate. After an incubation time of 18 h at 36°C the inhibition diameter was measured and the inhibition zone (distance between test disk and bacterial growth) was recorded.

### 2.3. Bone Cement

Antibiotic-containing PMMA bone cement and a PMMA bone cement reference (provided by Heraeus Medical GmbH, Wehrheim) were included in the tests. The bone cement was mechanically tested according to ISO 5833. The powder of one unit consisted of 40.0 g of polymer powder including zirconium dioxide as opaker and benzoyl peroxide. In addition, the powder contained the antibiotics listed in Table 1.

Test specimens were made of 5.0 g of powder and 2.5 ml of monomer. First a spatula was used to mix the two components; finally round specimens (*h* = 10.0 ± 0.1 mm, ⌀ = 25.0 ± 0.1 mm) were formed by using predefined forms.

### 2.4. Activity of Bone Cement Eluates against Planktonic Bacteria

Each of the three test specimens per cement was prepared as described above. The specimens were added to 10 ml of phosphate buffered saline (PBS; Sigma-Aldrich Chemie GmbH) and incubated at 36°C for 24 h. Thereafter the medium was removed and stored at −80°C until assayed. 10 ml of PBS was added again to the bone cement specimens. The procedure was repeated at 48, 72, and 96 h; however after 48 h, only 8 ml and after 72 h only 6 ml PBS were added.

The procedure for determining the antimicrobial activity of the eluates was as described for antibiotic solution. Instead of the antibiotic solution 10 *µ*l of the eluate was given on the test disc.

### 2.5. Activity of Bone Cement against One-Time Added Bacteria

Bone cement test specimens were placed in tubes. A suspension of 9.0 ml of nutrient broth (brain-heart-infusion broth; Oxoid) and of 1.0 ml of sheep blood was added for 20 min at 37°C. Following that, bacteria were added in a concentration of 10^6^ and 10^3^/10 ml, respectively. After 24 h of incubation at 37°C and mixing of the suspension, 10 *µ*l was taken and spread on TS-agar plates. Here, after 24 h of incubation at 37°C, the growth of bacteria was determined in a semiquantitative manner (no growth, up to 10 colonies, single colonies still visible, and confluent growth). The remaining broth was removed in a way that the test specimens were kept wet. 10 ml of nutrient broth per tube was added again and the tubes were incubated as before.

The procedure was analog after 48 h, 72 h, and 96 h; however at 48 h only 8 ml and at 72 h only 6 ml of broth were readded. This simulated the lower amount of wound fluid at these respective times. At 96 h, the experiment was finished. In one series each of the four test specimens was tested against 10^3^ and 10^6^ bacteria, respectively. Experiments were repeated; thus each of the eight single independent results was obtained.

### 2.6. Activity of Bone Cement against Repeated Added Bacteria

In this series including each of the five test specimens per cement and strain, bacteria were added in a concentration of 10^6^ at the beginning. In the following days, every 10^3^ of bacteria was added to the suspension again. The other procedure was made as described above.

### 2.7. SEM Photographs

Exemplarily, SEM photographs were taken after the one-time addition of* S. aureus* ATCC 44300 for 24 h. Samples were fixed in 2% glutaraldehyde in cacodylate buffer for 30 min, washed twice with cacodylate buffer, and dehydrated using a graded ethanol series (15 min for each concentration). Following critical point drying, samples were sputter-coated with gold and examined with a ZEISS LEO-1530 Gemini (Carl Zeiss NTS GmbH) equipped with a field emission electron gun at 7.5 keV.

### 2.8. Statistics

Most of the results are presented in a descriptive way. In addition, Chi^2^ test was used to compare results related to the different concentrations of the antibiotic as well as between the antibiotics (only the highest concentrations).

## 3. Results

### 3.1. MIC Values and Inhibition Zones of Antibiotics

The MIC values (determined in independent replicates with no difference between the two tests) were in the range between 0.125 *µ*g/ml and 128 *µ*g/ml for fosfomycin. The highest MIC was measured against* E. faecium *DSM 13590. MICs of daptomycin were 1 *µ*g/ml except for* E. faecium *DSM 13590 with 8 *µ*g/ml. The MICs of gentamicin varied between 0.25 *µ*g/ml (*S. aureus* ATCC 29213) and 64 *µ*g/ml (*S. aureus* ATCC 43300) (Table 2).

As expected, the inhibition zones of the antibiotics were related to the MIC values. Mostly, gentamicin did not increase the inhibition zones in comparison with single fosfomycin or daptomycin (Table 3).

### 3.2. Activity of Bone Cement Eluates

**Figure 1:**
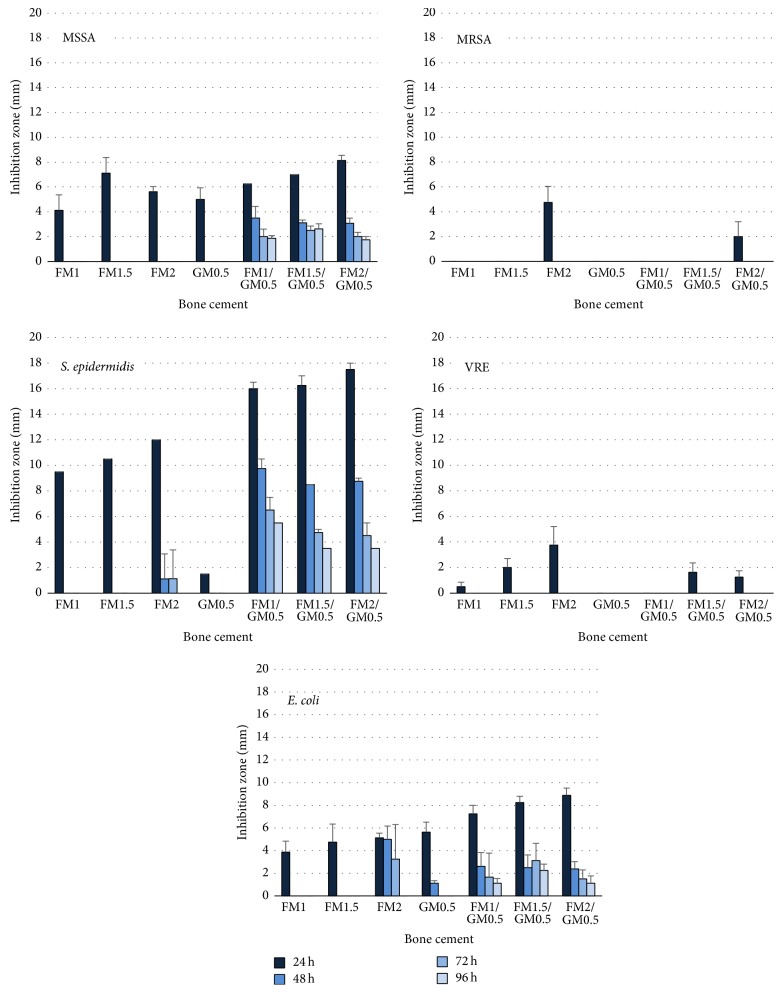
Inhibition zones (distance between test disk and bacterial growth, mm) of the eluates from 1.0 g, 1.5 g, and 2.0 g fosfomycin (FM), 0.5 g gentamicin (GM), and 1.0 g, 1.5 g, and 2.0 g fosfomycin/0.5 g gentamicin (FM/GM) containing bone cement obtained after 24 h, 48 h, 72 h, and 96 h against* Staphylococcus aureus* ATCC 29213 (MSSA),* S. aureus* ATCC 44300 (MRSA),* S. epidermidis* DSM 8913,* Enterococcus faecium *DSM 13590 (VRE), and* Escherichia coli* DSM 22311 (ESBL:SHV-1).

**Figure 2:**
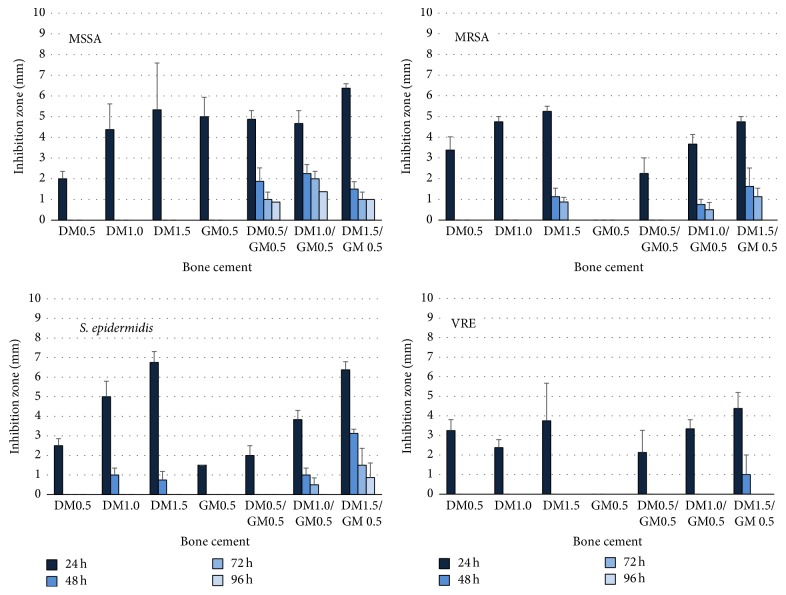
Inhibition zones (distance between test disk and bacterial growth, mm) of the eluates from 0.5 g, 1.0 g, and 1.5 g daptomycin (DM), 0.5 g gentamicin (GM), and 0.5 g, 1.0 g, and 1.5 g daptomycin/0.5 g gentamicin (DM/GM) containing bone cement obtained after 24 h, 48 h, 72 h, and 96 h against* Staphylococcus aureus* ATCC 29213 (MSSA),* S. aureus* ATCC 44300 (MRSA),* S. epidermidis* DSM 8913, and* Enterococcus faecium *DSM 13590 (VRE).

Relating the inhibition zones of bone cement eluates to those of the pure antibiotics there was an activity of up to 2-3 mg/ml fosfomycin after 24 h, it was decreasing to less than 0.5 mg/l after 48 h. The respective values for daptomycin were 0.2–0.4 mg/ml after 24 h and less than 0.05 mg/ml after 48 h. The addition of gentamicin prolonged antimicrobial activity of eluates of fosfomycin-containing cement against* S. aureus* ATCC 29213,* S. epidermidis* DSM 8913, and* E. coli* DSM 22311 and of daptomycin-containing cement against* S. aureus* ATCC 29213 and* S. epidermidis* DSM 8913 (Figures 1 and 2).

### 3.3. Bone Cement and One-Time Added Bacteria

Antibiotic-free bone cement never suppressed growth of any bacteria at any time-point.

**Figure 3:**
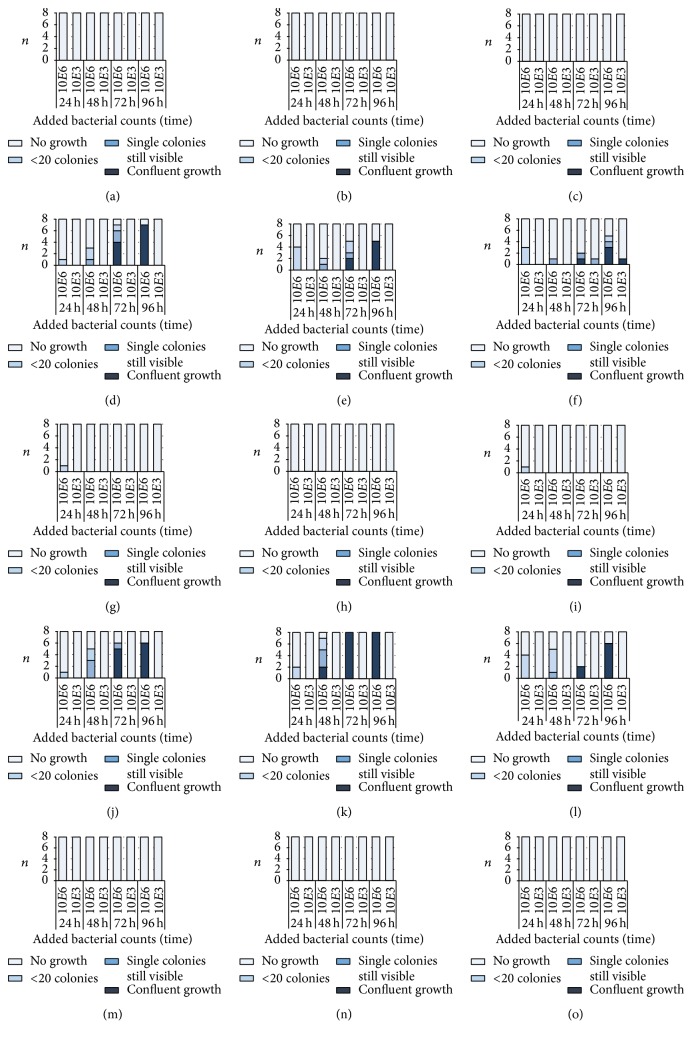
Number of samples (*n*) with the different growth of bacteria in the nutrient broth after incubation with test specimens of 1.0 g (a, d, g, j, m), 1.5 g (b, e, h, k, n), and 2.0 g fosfomycin (c, f, i, l, o)/0.5 g gentamicin-containing bone cement. Bacteria (*Staphylococcus aureus* ATCC 29213 (MSSA; (a), (b), (c)),* S. aureus* ATCC 44300 (MRSA; (d), (e), (f)),* S. epidermidis* DSM 8913 (g, h, i),* Enterococcus faecium *DSM 13590 (VRE; (j), (k), (l)), and* Escherichia coli* DSM 22311 (ESBL:SHV-1; (m), (n), (o))) were added at the beginning in a concentration of 10^3^  (10*E*3) or 10^6^  (10*E*6) to 10 ml of nutrient media. All controls (bone cement without antibiotics) showed confluent growth (not shown).

All fosfomycin-containing cement killed ultimately all* S. aureus* ATCC 23213 and* E. coli* DSM 22311 within 24 h, independently if 10^3^ or 10^6^ bacteria were added. After addition of 10^6^* S. epidermidis* DSM 8913 two test specimens remained positively with low bacterial counts after 24 h (1 (12.5%) with 0.5 g gentamicin + 1.0 g fosfomycin and 1 (12.5%) with 0.5 g gentamicin and 2.0 g fosfomycin); all other samples were negatively tested. When 10^3^* S. aureus* ATCC 43300 were added to fosfomycin-containing PMMA cement only one of all tested samples became positive after 72 h and 96 h. After addition of 10^6^* S. aureus* ATCC 43300 growth was suppressed up to 48 h; by tendency a concentration-depending activity of the antibiotic was found after 72 h; 7 (87.5%) tests were positive after 1.0 g fosfomycin and 2 (25%) after 2.0 g fosfomycin. No growth of* E. faecium* DSM 13590 was found when 10^3^ bacteria were added; however after 10^6^ bacteria 1–4 samples per group (12.5%–50%) were positive after 24 h and 75–100% after 72 h independent of the antibiotic concentration (Figure 3).

**Figure 4:**
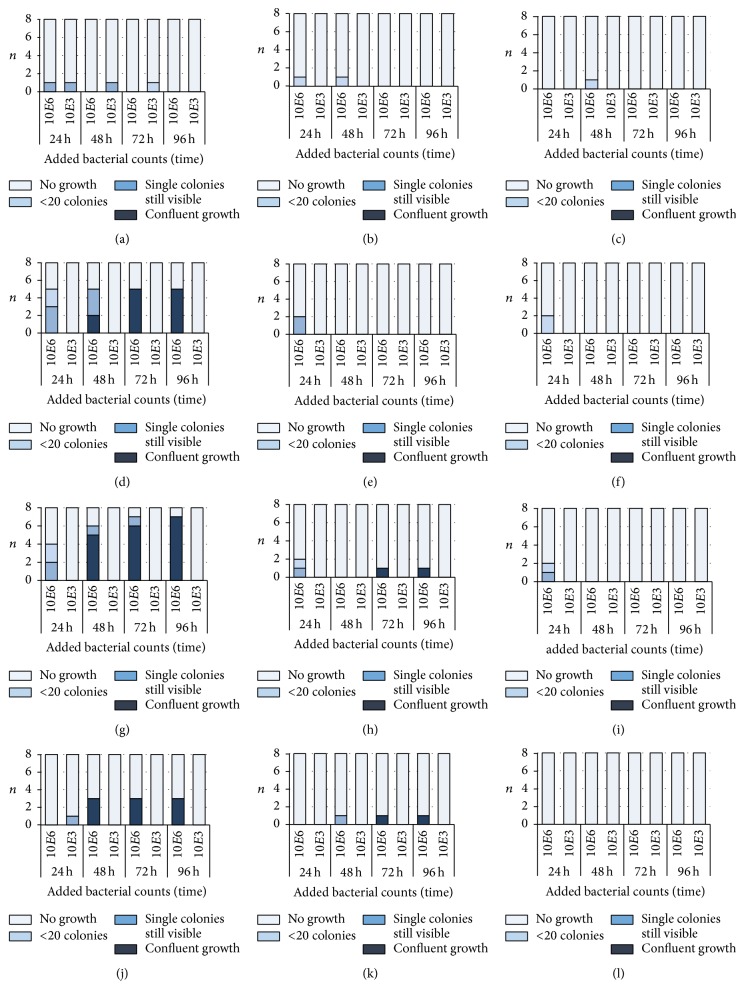
Number of samples (*n*) with the different growth of bacteria in the nutrient broth after incubation with test specimens of 0.5 g (a, d, g, j), 1.0 g (b, e, h, k), and 1.5 g daptomycin (c, f, i, l)/0.5 g gentamicin-containing bone cement. Bacteria (*Staphylococcus aureus* ATCC 29213 (MSSA; (a), (b), (c)),* S. aureus* ATCC 44300 (MRSA; (d), (e), (f)),* S. epidermidis* DSM 8913 (g, h, i), and* Enterococcus faecium *DSM 13590 (VRE; (j), (k), (l))) were added at the beginning in a concentration of 10^3^  (10*E*3) or 10^6^  (10*E*6) to 10 ml of nutrient media. All controls (bone cement without antibiotics) showed confluent growth (not shown).

After addition of bacteria to daptomycin-containing bone cement, all samples with 10^3^ of staphylococci and* E. faecium* DSM 13590 were completely negative or became negative during the experiment. When 10^6^ bacteria were added the finding was the same for* S. aureus* ATCC 29213. For the other included bacterial strains, the activity depended on the daptomycin concentration; 1.5 g daptomycin killed all bacteria whereas after 0.5 g daptomycin 3 (37.5%) to 7 (87.5%) samples were permanently positive at least from 48 h (Figure 4). Differences between the three concentrations of daptomycin were found for 10^6^ added* S. aureus* ATCC 43300,* S. epidermidis* DSM 8913 at 48 h (*p* = 0.013, *p* = 0.003), 72 h (*p* = 0.002, *p* = 0.003), and 96 h (*p* = 0.002, *p* < 0.001), and* E. faecium *DSM 13590 at 48 h (*p* = 0.032).

When comparing the highest concentration used of the fosfomycin-gentamicin-containing bone cement with those of the daptomycin-gentamicin-containing bone cement, daptomycin was more effective against* E. faecium* DSM 13590 (24 h: *p* = 0.021; 48 h: *p* = 0.026; 72 h: *p* = 0.002; 96 h: *p* = 0.002).

### 3.4. Bone Cement and Repeated Added Bacteria

**Figure 5:**
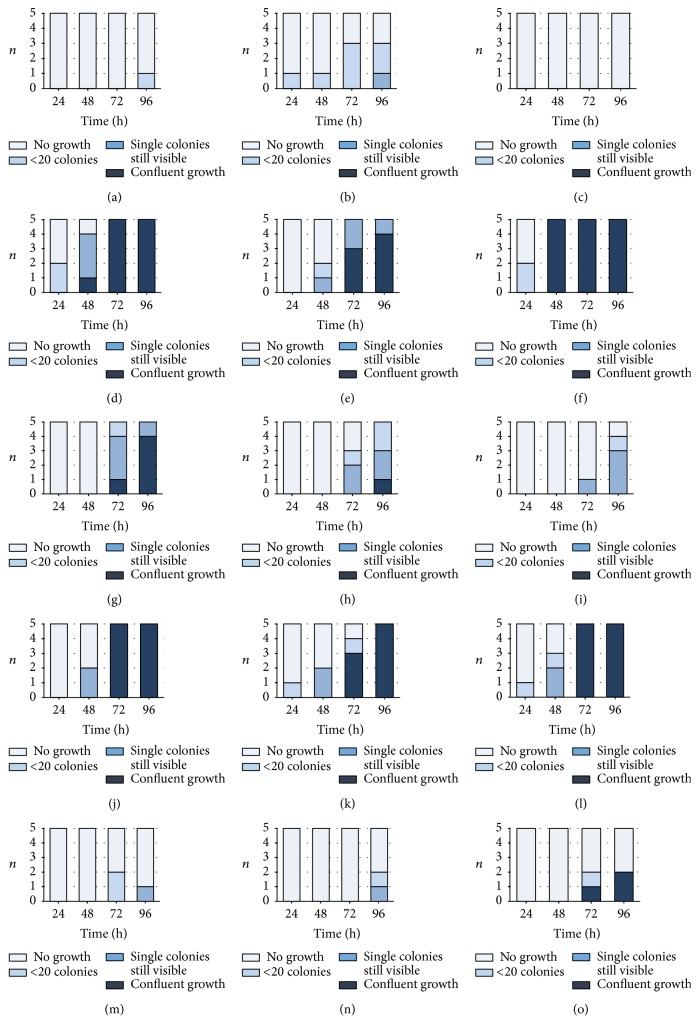
Number of samples (*n*) with the different growth of bacteria in the nutrient broth after incubation with test specimens of 1.0 g (a, d, g, j, m), 1.5 g (b, e, h, k, n), and 2.0 g fosfomycin (c, f, i, l, o)/0.5 g gentamicin-containing bone cement. Bacteria (*Staphylococcus aureus* ATCC 29213 (MSSA; (a), (b), (c)),* S. aureus* ATCC 44300 (MRSA; (d), (e), (f)),* S. epidermidis* DSM 8913 (g, h, i),* Enterococcus faecium *DSM 13590 (VRE; (j), (k), (l)), and* Escherichia coli* DSM 22311 (ESBL:SHV-1; (m), (n), (o))) were added at the beginning in a concentration of 10^6^/ml and from 24 h daily in a concentration of 10^3^. All controls (bone cement without antibiotics) showed confluent growth (not shown).

When bacteria were added repeatedly to fosfomycin-containing cement, growth of* S. aureus* ATCC 29213,* S. epidermidis* DSM 8913, and* E. coli* DSM 22311 was suppressed up to 96 h and that of* S. aureus* ATCC 43300 and of* E. faecium* DSM 13590 only up to 24 h (Figure 5).

**Figure 6:**
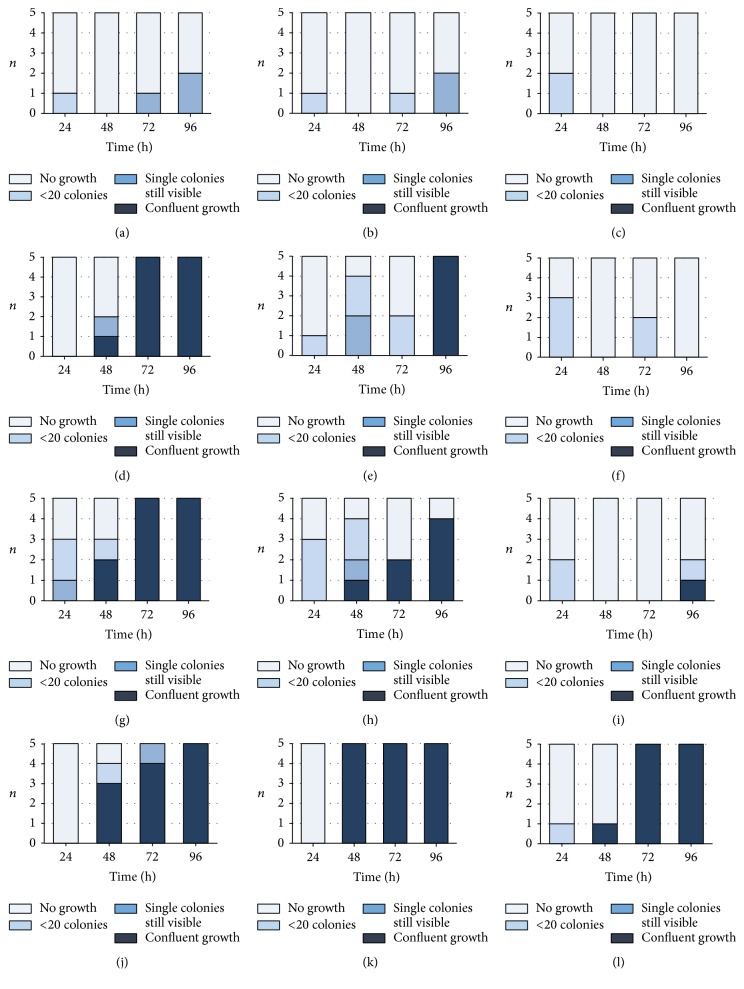
Number of samples (*n*) with the different growth of bacteria in the nutrient broth after incubation with test specimens of 0.5 g (a, d, g, j), 1.0 g (b, e, h, k), and 1.5 g daptomycin (c, f, i, l)/0.5 g gentamicin-containing bone cement. Bacteria (*Staphylococcus aureus* ATCC 29213 (MSSA; (a), (b), (c)),* S. aureus* ATCC 44300 (MRSA; (d), (e), (f)),* S. epidermidis* DSM 8913 (g, h, i), and* Enterococcus faecium *DSM 13590 (VRE; (j), (k), (l))) were added at the beginning in a concentration of 10^6^/ml and from 24 h daily in a concentration of 10^3^. All controls (bone cement without antibiotics) showed confluent growth (not shown).

An antibiotic concentration dependent activity was visible after repeated addition of bacteria to daptomycin-containing cement; growth was clearly suppressed by 1.5 g daptomycin. The activity on* E. faecium* DSM 13590 lasted 24 h independent of the daptomycin concentration. After 48 h there was only an activity by 1.5 g daptomycin and after 96 h, all samples became positive (Figure 6). Daptomycin concentration dependent differences were found for* S. aureus* ATCC 43300 at 72 h (*p* = 0.005) and 96 h (*p* = 0.001), for* S. epidermidis* at 72 h (*p* = 0.001), and for* E. faecium* DSM 13590 at 48 h (*p* = 0.043).

When comparing the highest concentration used of the fosfomycin-gentamicin-containing bone cement with those of the daptomycin-gentamicin-containing bone cement, those specimens with daptomycin were more effective against* S. aureus *ATCC 43300 at 48 h (*p* = 0.002), at 72 h (*p* = 0.007), and at 96 h (*p* = 0.002).

### 3.5. SEM Photographs

**Figure 7:**
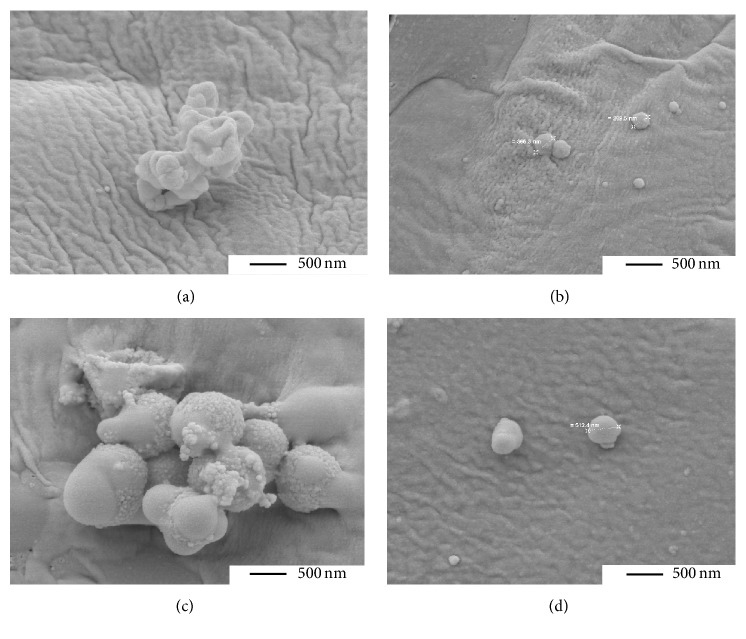
SEM photographs (20,000-fold magnification) of fosfomycin-containing bone cement (1.0 g fosfomycin/0.5 g gentamicin (a, b)) and of daptomycin-containing bone cement (0.5 g daptomycin/0.5 g gentamicin (c); 1.5 g daptomycin/0.5 g gentamicin (d)) with* S. aureus* ATCC 44300 after 24 h of incubation.

SEM photographs confirmed the results for* S. aureus* ATCC 43300. Bacteria were clearly damaged; however often they were of limited size in the presence of bone cement containing fosfomycin or daptomycin (Figure 7).

## 4. Discussion

The developing antibiotic resistance implicates the search for new therapeutic strategies. Preliminary experiments using the same method as in the current study showed no activity of bone cement containing gentamicin only against MRSA strains including* S. aureus* ATCC 43300; a confluent growth of bacteria was observed already every 24 h after addition of the bacterial strains even in a concentration of 10^3^ bacteria/10 ml. In this in vitro study, the activities of fosfomycin/gentamicin and daptomycin/gentamicin-loaded bone cement as potential alternatives were tested against selected bacterial strains. Mostly an alternative antibiotic is combined with gentamicin. In vitro a longer lasting activity is measured, for example, for combinations of vancomycin or teicoplanin with gentamicin in comparison with the single antibiotic [15].

The main focus of this in vitro study was a potential antibiofilm activity of the bone cement. The clinical situation immediately after surgery was imitated in two different ways. In a first series, bone cement was exposed only one time to bacteria mimicking a local infection, where infected tissues are removed surgically and the remaining residing bacteria were to be killed by the antibiotic. In the second series, a constant spreading of bacteria in low numbers was suggested in addition. The test specimens were always placed in nutrient broth with 10% of sheep blood allowing coating with a protein layer and thereafter biofilm formation. In all samples with antibiotic-free test specimens a strong biofilm formation was seen. The media were exchanged daily with decreasing volumes of fresh media.

Fosfomycin, a phosphonic acid derivative, is a naturally occurring antibiotic which displays a broad-spectrum activity against Gram-positive and Gram-negative bacteria by inhibiting cell wall synthesis [16]. It is highly active against MSSA, MRSA, and* E. coli* and exerts an intermediate activity to VRE [17]. It is in clinical use in soft tissue infections, osteomyelitis, abdominal infections, and urinary tract infections [16]. Reports about fosfomycin as an additive to resins to be used in orthopedic surgery are rare. Several years ago, addition of fosfomycin to a biodegradable composite was suggested as treatment option [18]. Recently fosfomycin combined with gentamicin was added to PMMA powder; eluates from these cement types were highly active against MRSA [19]. In the present study, fosfomycin exerted an excellent activity against MSSA,* S. epidermidis*, and* E. coli* strains. The used PMMA bone cement contained gentamicin in addition to fosfomycin; the antimicrobial activity of the eluate was higher and lasted longer against strains being sensitive to both antibiotics when compared to fosfomycin alone. In a foreign body animal model systemic applied fosfomycin was highly active against MRSA, but complete biofilm elimination was only achieved when combined with rifampicin [20]. In our assays with bone cement test specimens, an activity depending on the bacterial concentration was found; low counts of bacteria were killed.

Daptomycin is a cyclic lipopeptide which targets Gram-positive membranes by formation of ring-like pores [21]. It is highly active against* S. aureus*, coagulase-negative staphylococci,* Enterococcus faecalis*, and* E. faecium*; during an eight-year surveillance no significant increase or region variation of resistance was observed [22]. In clinical trials, daptomycin was found to be equal with standard antibiotics in severe skin infections, bacteremia, and endocarditis caused by Gram-positive bacteria [23]. A case report documented a successful treatment of a recurrent prosthetic hip infection in a 70-year-old female with a past history of MRSA infection by using two-stage revision surgery with PMMA impregnated with daptomycin and gentamicin [24].

As for fosfomycin, daptomycin was highly active against a low count of bacteria. Both tests with eluates as well as with bone cement specimens revealed that lowest concentrated daptomycin (0.5 g/40 g powder) was not as active as the highest tested concentration of the antibiotic (1.5 g/40 g powder) against MRSA,* S. epidermidis,* and* E. faecium*. This may be confirmative to a recent reports about release profiles of PMMA bone-cement showing a continuous longer release [25–27] and antimicrobial activity of daptomycin when loaded with higher concentrations [26, 27]. If the cement was loaded with daptomycin and gentamicin, release profiles showed a prolonged antimicrobial activity in comparison with the single antibiotics. Daptomycin combined with gentamicin exerted synergistic activity on intracellular* S. aureus* located intracellularly in osteoblasts [28]. An increased in vitro antibiofilm activity of daptomycin together with gentamicin in comparison with daptomycin only was reported recently [29]. This only other in vitro study focusing on antibiofilm activity of daptomycin-containing cement underlines an efficacy at least for 72 h when inoculated with 10^6^* S. epidermidis* [29].

A higher antibacterial activity was found for the cement containing daptomycin/gentamicin than for those containing fosfomycin/gentamicin against Gram-positive bacteria. Opposite to fosfomycin-containing cement, the used highest concentrated daptomycin-containing cement clearly suppressed the growth of the one-time added MRSA and VRE strains up to 96 h. When the strains were added repeatedly, daptomycin cement specimens showed a more prolonged activity against the MRSA strain. However, as daptomycin is inactive against Gram-negatives, fosfomycin might be a treatment opportunity against ESBL strains which should be verified in follow-up studies. The dependence of the activity of fosfomycin/gentamicin and daptomycin/gentamicin-containing bone cement on the bacterial strain may underline a need for rapid microbiological diagnostics, for example, by nucleic-acid based methods for rapid detection of ESBL [30] and VRE [31] directly from clinical specimens, or as described for identifying methicillin-resistant staphylococci in prosthetic joint infections [32].

The tested PMMA bone cement was active against low numbers of bacteria where biofilm formation was clearly inhibited. This underlines necessity of mechanical debridement of the wound area. Only an activity on biofilm formation can be anticipated. Experiments on established biofilms showed biofilm eradication concentrations of* S. aureus* and* S. epidermidis* biofilms as higher as 1024 *µ*g/ml daptomycin recently [33]. The high resistance of bacteria within biofilms is related to the slow or incomplete penetration of the matrix, the physiologic response, for example, gene transfer, and the development of persister cell populations [34]. The small size of different* S. aureus* cells after contact with PMMA bone cement in SEM photographs may indicate the development of persisters under antibiotic pressure. This well-known phenomenon should not be neglected; it was recently shown for* S. aureus* cells when exposed to 10-fold MIC of daptomycin [35].

This in vitro study indicates daptomycin-containing PMMA bone cement as a potential alternative in Gram-positive prosthetic joint infections, whereas fosfomycin-containing cement might be helpful in infections with ESBL strains. Our study covered mainly aspects of suppression of bacterial growth (antibiofilm activity) in the presence of bone cement specimens considering a different volume of wound fluid. Upcoming research should address in more detail the release kinetics of the antibiotics from the cement, a potential synergistic activity of the antibiotics, and a potential development of resistance (persisters).

## 5. Conclusion

In summary, PMMA bone cement with higher concentrations of daptomycin (1.5 g/40 g powder) may be an alternative in treatment of selected periprosthetic infections by Gram-positives. However follow-up research is necessary to evaluate in more detail the release kinetics of the antibiotics, synergistic activity of the antibiotics, the possible development of resistance (persisters), and the potential of fosfomycin-containing PMMA cement to target ESBL strains.

## Figures and Tables

**Table 1:** Antibiotic content per unit test cement (40.0 g).

Cement	Gentamicin content [g]	Fosfomycin content [g]	Daptomycin content [g]
1	0.5	1.0	—
2	0.5	1.5	—
3	0.5	2.0	—
4	0.5	—	0.5
5	0.5	—	1.0
6	0.5	—	1.5
7	0.5	—	—
8	—	—	—
9^1^	0.5	—	—
10^1^	—	1.0	—
11^1^	—	1.5	—
12^1^	—	2.0	—
13^1^	—	—	0.5
14^1^	—	—	1.0
15^1^	—	—	1.5

^1^These cement types were only included in experiments determining the activity of bone cement eluates.

**Table 2:** Minimal inhibitory concentrations of antibiotics against test strains.

	Gentamicin (*µ*g/ml)	Fosfomycin (*µ*g/ml)	Daptomycin (*µ*g/ml)
*S. aureus* ATCC 29213 (MSSA)	0.25	4	1
*S. aureus* ATCC 43300 (MRSA)	64	8	1
*S. epidermidis* DSM 8913	8	0.125	1
*E. faecium* DSM 13590 (VRE)	16	128	8
*E. coli* DSM 22311 (ESBL:SHV-1)	2	4	

**Table 3:** Inhibition zones antibiotics (distance (mm) between test disk and bacterial growth).

Strain	GM (*µ*g/disc)	Only GM	Fosfomycin (*µ*g/disc)				Daptomycin (*µ*g/disc)			
			3.9	7.8	15.6	31.2	2	5.9	7.8	23.4
*S. aureus* ATCC 29213	0	0	0	4	6	7	5	6.5	7.5	8.5
	2	6	3.5	4	n.d.	n.d.	4.5	5	n.d.	n.d.
	7.8	8.5	n.d.	n.d.	6	7	n.d.	n.d.	5.5	6.5

*S. aureus* ATCC 43300	0	0	0	0	0	4.5	5.5	6.5	7.5	8.5
	2	0	0	0	n.d.	n.d.	1	2	n.d.	n.d.
	7.8	0	n.d.	n.d.	1	2	n.d.	n.d.	2.5	4.5

*S. epidermidis *DSM 8913	0	0	5	7.5	10	13.5	7	7.5	9	10
	2	0	13.5	15.5	n.d.	n.d.	4.5	7	n.d.	n.d.
	7.8	0	n.d.	n.d.	19.5	22	n.d.	n.d.	7	8

*E. faecium* DSM 13590	0	0	0	0	1	2.5	3	4	5	6.5
	2	0	0	8	n.d.	n.d.	0	4	n.d.	n.d.
	7.8	1	n.d.	n.d.	0	10	n.d.	n.d.	2	5

*E. coli* DSM 22311	0	0	6	8.5	9.5	12	n.d.	n.d.	n.d.	n.d.
	2	6	3	4	n.d.	n.d.	n.d.	n.d.	n.d.	n.d.
	7.8	9.5	n.d.	n.d.	5.5	12	n.d.	n.d.	n.d.	n.d.

n.d.: not done; GM: gentamicin.

Discs of fosfomycin 31.2 *µ*g/gentamicin 7.8 *µ*g and fosfomycin 7.8 *µ*g/gentamicin 2 *µ*g mimicking cement 2 g fosfomycin/0.5 g gentamicin.

Discs of fosfomycin 15.6 *µ*g/gentamicin 7.8 *µ*g and fosfomycin 3.9 *µ*g/gentamicin 2 *µ*g mimicking cement 1 g fosfomycin/0.5 g gentamicin.

Discs of daptomycin 23.4 *µ*g/gentamicin 7.8 *µ*g and daptomycin 5.9 *µ*g/gentamicin 2 *µ*g mimicking cement 1.5 g daptomycin/0.5 g gentamicin.

Discs of fosfomycin 7.8 *µ*g/gentamicin 7.8 *µ*g and fosfomycin 2 *µ*g/gentamicin 2 *µ*g mimicking cement 0.5 g daptomycin/0.5 g gentamicin.
